# Blood pressure and heart rate during ovariohysterectomy in pyometra and control dogs: a preliminary investigation

**DOI:** 10.1186/s13028-016-0263-y

**Published:** 2016-11-17

**Authors:** Odd Viking Höglund, Johanna Lövebrant, Ulf Olsson, Katja Höglund

**Affiliations:** 1 Department of Clinical Sciences, Box 7054, 750 07 Uppsala, Sweden; 2 Alingsås Veterinärstuga AB, Björkekärrsvägen 26, 441 91 Alingsås, Sweden; 3 Unit of Applied Statistics and Mathematics, Swedish University of Agricultural Sciences, Box 7032, 750 07 Uppsala, Sweden; 4 Department of Anatomy, Physiology and Biochemistry, Swedish University of Agricultural Sciences, Box 7011, 750 07 Uppsala, Sweden

**Keywords:** Surgical stress, Blood pressure, Heart rate, Canine, Pyometra, Isoflurane, Acepromazine, Methadone

## Abstract

**Background:**

Surgery causes a stress response, a physiologic response to trauma. The intraoperative surgical stress response in dogs diagnosed with pyometra has not previously been described. The aim of this study was to investigate the intraoperative surgical stress response, assessed by blood pressure and heart rate measurements, in dogs diagnosed with pyometra and healthy controls. All dogs were premedicated with acepromazine and methadone, anaesthesia was induced with propofol and maintained with isoflurane, where after the dogs were subjected to ovariohysterectomy.

**Results:**

Eight dogs diagnosed with pyometra and eight healthy controls were used. Systolic blood pressure and heart rate were measured to assess the surgical stress response. Additionally propofol dosage at induction of anaesthesia and the end-tidal isoflurane concentration were investigated. The surgery was split into four phases. Phase 0 was the period 10 min before the skin incision, phase 1 was skin incision and opening of abdomen, phase 2 was manipulation of uterine horns, lifting of the ovary with stretching of the mesovarium, ligation and transection of mesovarium and phase 3 was ligation and transection of cervix, removal of organs and closing of the abdomen. Dosage of propofol at induction of anaesthesia was 3.6 ± 1 mg/kg in dogs with pyometra and 4.1 ± 1 in healthy controls (*P* = 0.37). In both groups, systolic blood pressure increased between phase 1 and 2, from 87 ± 15 to 114 ± 19 mmHg in dogs with pyometra, and from 88 ± 18 to 106 ± 20 mmHg in healthy controls, (both *P* < 0.0001). Systolic blood pressure did not differ significantly between groups in any of the phases. Heart rate and end-tidal concentration of isoflurane did not differ significantly between phases or between groups.

**Conclusions:**

The increased blood pressure at removal of ovaries during ovariohysterectomy suggests a pronounced noxious stimulus at this part of the procedure. In principle, the study parameters and response to surgery did not differ significantly between dogs with pyometra and healthy controls.

## Background

Pyometra is a common disease in intact bitches. In a study of insured dogs in Sweden approximately 25% of females were affected by pyometra before 10 years of age [1]. Protective and risk-factors may vary between breeds as there are differences in incidence rates between breeds [2–5]. Surgery is curative of the disease. In animals subjected to surgery, the noxious stimuli caused by the procedure triggers a stress response [6–8] which can be used for assessment and evaluation of surgical methods and anaesthetic protocols [9–14]. The stress response is a physiological response to trauma or surgery and is generally considered to be proportional to the degree of surgical trauma [15–18]. Physiological parameters such as heart rate and blood pressure can be used as surrogate measure of the stress response and there are studies of the perioperative surgical stress response in healthy female dogs subjected to neutering [11, 19–27]. However, the effect of pyometra on the intraoperative surgical stress response is not known. It may be expected that patients affected by pyometra and endotoxemia [28–33] will differ in intraoperative hemodynamic response compared to healthy dogs due to the effects on circulation of the endotoxins [34–36]. Based on previous studies, we hypothesized that noxious stimuli at removal of ovaries would cause systolic blood pressure to peak, regardless if pyometra was diagnosed or not. The aim of this study was to investigate the intraoperative surgical stress response, assessed by blood pressure and heart rate measurements, in dogs diagnosed with pyometra and healthy controls, all of which were subjected to ovariohysterectomy (OHE).

## Methods

### Animals

The study population consisted of female privately owned dogs admitted to the University animal hospital for surgical treatment, OHE, due to pyometra. Dogs of different breeds and ages were included (Table 1). All dogs were examined by an experienced veterinarian and diagnosis was confirmed with ultrasound and/or radiographs. The control group consisted of eight female privately owned dogs that were confirmed healthy by owner interview and clinical examination and that were admitted for elective OHE at the owners’ request (Table 1). The Uppsala Animal Ethics Committee, Sweden and Swedish Board of Agriculture approved the study (C 127/10).

**Table 1:** The age and body weight of the 16 dogs included in the study and duration of phases (mean ± standard deviation)

Dogs	n	Age (years)	Weight (kg)	Phase 0 (min)	Phase 1 (min)	Phase 2 (min)	Phase 3 (min)
Pyometra	8	5.4 ± 2	22.7 ± 13	10 ± 0	6.9 ± 5	14.0 ± 6	16.6 ± 6
Controls	8	4.7 ± 2	17.5 ± 7	10 ± 0	7.8 ± 4	12.9 ± 4	20.0 ± 8

Age, bodyweight and duration of phases did not differ between groups (Student’s *t* test, *P* > 0.05)

### Anaesthesia

Acepromazine and methadone were used for sedative premedication (Table 2). Carprofen was given preoperatively or immediately at end of surgery. Postoperatively, methadone hydrochloride was administered subcutaneously every 6 h for the first 24 h.

**Table 2:** Drugs used for premedication and anesthesia of the 16 dogs included in the study

Drug	Indication	Dosage	Trade name	Route
Acepromazine	Sedative	0.03 mg/kg	Plegicil vet, Vericore Ltd	Intramuscular
Carprofen	Analgesic	4 mg/kg	Rimadyl vet, Pfizer ApS	Subcutaneous
Methadone hydrochloride	Analgesic	0.2 mg/kg	Metadon Recip, Recip AB	Subcutaneous
Propofol	General anesthesia	To effect	PropoVet Multidose, Orion Pharma Animal Health	Intravenous
Isoflurane	General anesthesia	To effect	Isoba vet, Schering-Plough A/S	Inhalation

Anaesthesia was induced by intravenous administration of propofol (PropoVet Multidose, 10 mg/ml, Orion Pharma Animal Health) given to effect at approximately 4 mg/kg and maintained with inhalation of isoflurane mixed in air at 2–3%. Oxygen and air mixture at approximately 50% was used at a flow of approximately 20–40 ml/kg/min in a circle breathing system. The dosage of isoflurane was controlled by an experienced and qualified veterinary nurse who monitored the anaesthesia. All dogs breathed spontaneously. The setting of the vaporizer was aimed at maintaining the dog anesthetized at surgical plane of anaesthesia (stage 3, plane 2). Depth of anaesthesia was judged by subjective assessment of the muscular tonus of the jaw, position of the eyeballs, dilation of the pupils, and the response to surgery.

The end-tidal isoflurane concentration heart rate and blood pressure were recorded using a multi-parameter anaesthetic monitoring device (LifeWindow 6000, Digicare Biomedical Technology, Boynton Beach, FL, USA). Non-invasive arterial blood pressure and heart rate were monitored by use of an oscillometric method. An automatic pneumatic cuff, with a width to circumference ratio of approximately 0.4, was wrapped around the distal forelimb and connected to the monitoring device. Data were registered once per minute following induction of anaesthesia until completion of surgery. Data was transferred to an Excel file (Microsoft Office) and all data points were included in the analysis.

### Surgery

The dog was placed in dorsal recumbency and the animal´s abdomen was clipped and scrubbed. A ventral midline incision 2–3 cm cranial to the umbilicus was made and extended caudally and the abdomen was opened. The uterus was located, the ovaries were identified and a hole was made in the mesovarium. Haemostat forceps were placed across the ovarian pedicle. Forceps were repositioned distally (closer to the ovary) to expose the compressed mesovarial tissue and one or two ligatures were placed and tied. An absorbable suture size 2–0 or 3–0 was used for ligation. The ovarian pedicle was cut between the distal ligature and the forceps. A ligature was also placed over the broad ligament. The procedure was repeated on the contralateral side. The uterine body was ligated at the cranial tip of the cervix, the tissue was transected and the uterus was removed. The ligated tissue was observed and verified for hemostasis. The abdominal wall was closed in three layers (*linea alba*, subcutaneous tissue and skin).

### Protocol for data analysis

A protocol was designed for analysis of the different parts of the surgery. For comparison and evaluation of the cardiovascular response the surgery was split into four phases: 10 min immediately before skin incision (phase 0), skin incision and opening of abdomen (phase 1), manipulation of uterine horns, ligation and transection of mesovarium (phase 2), ligation of cervix, removal of organs and closing (phase 3). Other actions by the surgeon that could affect the surgical stress response were noted in the protocol, i.e. application of forceps or diathermy. The exact time when all parts of the surgical procedures that represented each phase were performed were noted in the protocol. The recorded data was thus labelled by phase.

### Statistics

Student’s *t* test (two-sided, unequal variance) was used (Excel, Microsoft Office) for comparison of age, body weight, dosage of propofol and duration of phases between the groups (Tables 1, 5). Remaining statistical analysis was performed with the statistical program SAS (Cary, NC, USA). No serious deviations from normality were detected. The effects of surgery on heart rate, systolic blood pressure and end-tidal concentration of isoflurane in each phase were analysed using mixed linear models for repeated measures data [37, 38]. The least square means (LSMean) and standard error of least square means were computed and compared within and between groups (Procedure mixed) for each phase. Significant differences were calculated comparing LSMean with Tukey–Kramer’s adjustment for multiplicity. Results are reported as mean and standard deviation (SD) of each phase (Tables 3, 4, 5). *P* < 0.05 was considered significant for the analyses.

**Table 3:** Systolic blood pressure (Mean ± standard deviation, mmHg) in control dogs and dogs diagnosed with pyometra that were subjected to ovariohysterectomy

Diagnosis	n	Phase 0	Phase 1	Phase 2	Phase 3
Pyometra	8	87.1 ± 17	86.8 ± 15	114.5^a,b^ ± 19	101.2 ± 17
Control	8	77.6 ± 12	88.5 ± 18	105.6^a,b^ ± 20	98.8^a^ ± 21

Data was compared by the mixed procedure (SAS) with Tukey–Kramer’s adjustment (*P* < 0.05). There were no significant differences between the groups’ corresponding phases

^a^Differ significantly compared with phase 0

^b^Differ significantly compared with previous phase

**Table 4:** Heart rate (Mean ± standard deviation, beats per minute) in control dogs and dogs diagnosed with pyometra that were subjected to ovariohysterectomy

Diagnosis	n	Phase 0	Phase 1	Phase 2	Phase 3
Pyometra	8	95.3 ± 18	91.8 ± 17	106.8 ± 15	106.5 ± 15
Control	8	89.5 ± 22	91.5 ± 22	89.6 ± 20	95.2 ± 19

Data was compared by the mixed procedure (SAS) with Tukey–Kramer’s adjustment (*P* < 0.05). There were no significant differences compared to previous phase or between the groups’ corresponding phases

**Table 5:** Dosage of propofol (mg/kg) and end‐tidal concentration of isoflurane, % (Mean ± standard deviation) in control dogs and dogs diagnosed with pyometra that were subjected to ovariohysterectomy

Diagnosis	n	Propofol induction	Isoflurane phase 0	Isoflurane phase 1	Isoflurane phase 2	Isoflurane phase 3
Pyometra	8	3.6 ± 1	1.9 ± 0.3	1.8 ± 0.3	2.1 ± 0.3	2.0 ± 0.2
Control	8	4.1 ± 1	2.2 ± 0.3	2.0 ± 0.2	2.1 ± 0.2	1.9 ± 0.2

Dosage of propofol was compared between groups with student’s *t* test (*P* = 0.37). Data for end‐tidal concentration of isoflurane was compared by the mixed procedure (SAS) with Tukey–Kramer’s adjustment (*P* < 0.05). There were no significant differences compared to previous phase or between the groups’ corresponding phases

## Results

Age and body weight (Table 1) did not differ significantly between the groups (*P* = 0.6 and 0.3, respectively). There were no significant differences between the groups’ corresponding phases in systolic blood pressure, heart rate or concentration of anaesthetic gas. Duration of phases 0–3 for pyometra and control dogs did not differ significantly between groups (*P* = 1.0, 0.7, 0.7 and 0.3 respectively; Table 1).

The values for systolic blood pressure, heart rate and end-tidal concentration of isoflurane are given in Tables 3, 4 and 5, respectively. In both groups the systolic blood pressure (Table 3) did not change significantly at opening of the abdomen (phase 0–1) but increased between phase 1 and 2 (both *P* < 0.0001). Neither heart rate, nor end-tidal concentration of isoflurane, changed significantly during the procedures in any of the groups (Tables 4, 5). Dosage of propofol was 10% below the manufacturer’s recommended dosage (4 mg/kg) in the dogs with pyometra. The dosage did not differ significantly between the groups (*P* = 0.37; Table 5).

The surgeries were performed in total by seven different surgeons whereof two surgeons were involved in the control group and six in the pyometra group. In both groups, the ovarian pedicle was single ligated in six dogs and was double ligated in two dogs at removal of ovaries.

One dog in the pyometra group was given carprofen before surgery whereas six dogs were given carprofen immediately at end of the surgery. Of the control dogs seven dogs were given carprofen before surgery whereas one dog was given carprofen immediately at end of surgery. One dog in the pyometra group was on meloxicam treatment, no NSAID was given to that dog at day of surgery.

### Complications

In one dog in the control group an intraoperative haemorrhage from a single ligated ovarian pedicle was observed. The ovarian pedicle was swiftly re-ligated, haemostasis was confirmed and blood loss was not considered significant.

In another control dog a postoperative haemorrhage was suspected. The dog was re-anesthetized, the abdomen was opened and haemorrhage was observed from the left ovarian pedicle which had been single ligated. The ovarian pedicle was re-ligated, the abdomen closed and the dog recovered uneventfully.

One dog in the control group developed diarrhoea after 2 days on carprofen and the drug was discontinued. In another dog in the control group a wound infection was suspected. Antibiotics were prescribed and the dog recovered uneventfully. All dogs in the pyometra group recovered uneventfully.

## Discussion

In this study, the intraoperative surgical stress response in dogs diagnosed with pyometra and controls was assessed by measurements of systolic blood pressure and heart rate. The response did not differ significantly between groups. In both groups there was a peak of blood pressure at removal of ovaries which is in agreement with other studies of surgical stress in dogs subjected to OHE [11, 19, 25–27]. This part of the procedure, ligation of the mesovarium and removal of ovaries, caused a significant increase in blood pressure, regardless if the dogs were diagnosed with pyometra or not. Removal of ovaries is considered maximum noxious stimuli during a bitch spay [11, 25]. Attempts to reduce the noxious stimuli at removal of the ovaries by adding local anaesthetic to the mesovarium has been investigated with no proven benefit in dogs [21], while a positive effect was reported in a study in cats [39]. As results of these previous studies on local anaesthesia of the mesovarium are in conflict, further studies on targeted analgesia are needed. Less surgical stress at ovary removal has been reported in healthy dogs with a laparoscopic technique compared to the traditional open abdomen technique [11] and a laparoscopic-assisted technique for pyometra is described [40–42]. Furthermore, development of a new method for ligation of the mesovarium may enable a more standardized technique with potentially less noxious stimuli [43–45].

Most drugs used in the present study, except carprofen, may cause hypotension [46–48] and dogs of both groups could be considered hypotensive [49]. Acepromazine is a neuroleptic substance that blocks the post-synaptic dopamine receptors in the central nervous system (CNS). Methadone is an opiate agonist (µ-receptor agonist) and one of the potential adverse effects of methadone is bradycardia, which was an unlikely side effect at the dosage used and was not observed in the present study. The half-life of methadone is 10.7 ± 4.3 h following subcutaneous (s.c.) administration, and half-life of acepromazine is 7.1 ± 1.3 h following intravenous (i.v.) administration [50, 51]. There may have been some variation in time between premedication and induction of anaesthesia, but this variation was assumed to be small in comparison to the half-lives of the drugs. It can therefore be assumed that the intraoperative effects of the drugs were similar in both groups considering the half-lives of these drugs. The end-tidal concentration of isoflurane was stable throughout the procedures but could be considered high. A high or low isoflurane concentration has not been shown to have any effect on magnitude of blood pressure increase after stimuli in humans [47]. In another study on people, different concentrations of desflurane did not influence the endocrine response to surgery [52]. In the present study the end-tidal isoflurane concentration did not differ significantly between phases or the groups’ corresponding phases.

Carprofen is a weak cyclo-oxygenase (COX) inhibitor and the mechanism behind its main anti-inflammatory effect is unknown. No perioperative adverse effects on the cardiovascular system have been reported at recommended dosages [53, 54]. The usual procedure at the University animal hospital is to administer NSAID to patients affected by pyometra immediately after surgery, and not before. In contrast, the majority of the control dogs were administered NSAID before surgery. The treatment of the two groups with carprofen therefore differed and comparison between the two groups should be made cautiously.

In a previous study where markers of surgical stress in dogs subjected to OHE were measured, the heart rate increased at ligation of mesovarium and removal of the ovary [25]. The same anaesthetic protocol was used in the two studies but heart rate did not increase in the present study. However, the phases were defined differently and comparisons should be made with care. In the previous study removal of one ovary represented one phase, whereas in the present study removal of both ovaries represented one phase. Removal of ovaries in the present study caused blood pressure to increase, however, the longer duration for removal of both ovaries may have allowed the baroreceptor reflex to normalise a possible initial increase in heart rate.

In one dog an intraoperative haemorrhage was observed after single ligation of the ovarian pedicle. In addition one dog was re-operated due to haemorrhage from a single ligated ovarian pedicle. The complications described in this study confirm the current recommendation that the ovarian pedicle should be double ligated [55].

There were study limitations. One limitation was the use of non-invasive blood pressure measurements. An invasive technique is considered gold standard, but with repeated measurements a reliable average of a series of systolic blood pressure readings can be obtained. Systolic blood pressure values are given as the non-invasive method may be less accurate at lower pressures [56–58]. The use of systolic blood pressure values therefore lowers the risk of measurement errors. The number of dogs used in this study was low, individual variations may have affected the results, and therefore the results should be interpreted cautiously. Another variable that could have influenced the results was the dogs’ general condition. Possible endotoxemia was not investigated. Dogs with pyometra may have been affected by endotoxins which have effect on circulation [34–36]. The dogs’ status of pain, their blood pressure or the possible influence of endotoxins before surgery may have varied between dogs. No measurements of heart rate or blood pressure or scoring of pain were made before or after surgery. However, there were no significant differences between the groups’ intraoperative study parameters at corresponding phases and duration of phases did not differ between groups. Furthermore, pyometra dogs may have been given crystalloid fluids i.v. preoperatively and the exact timing is not known, which is an additional study limitation. However, if fluids were administered preoperatively, the effect on study parameters was considered to be minor as administration of isotonic fluids has little effect on arterial blood pressure according to a study on healthy dogs [59]. Extrapolation of results to dogs with concurrent disease should be done cautiously. There were seven different surgeons involved, which may have caused disparity in tissue handling and surgical technique. However, in a previous study no effect was seen on blood pressure changes of covariate surgeon [11]. This was considered to be in agreement with a study which demonstrated no effect of surgeon experience and duration of surgery on post-operative pain [60]. The person handling the anaesthesia was not aware of study aim or study parameters, but was not blinded to diagnosis, which may be considered a study limitation. The judgment of depth of anesthesia is partly subjective and deep planes of anesthesia may have been a reason for the observed low blood pressure. End-tidal concentration of isoflurane did not change significantly and did not differ between groups, but dosage requirements of end-tidal isoflurane concentration may differ between individuals. Furthermore, variations in carbon dioxide levels and body temperatures may affect the cardiovascular system. Neither of these parameters were evaluated, which is another study limitation.

## Conclusions

In principle, the study parameters and the response to surgery did not differ significantly between corresponding phases of the groups. Blood pressure increased at removal of the ovaries, both in dogs diagnosed with pyometra and healthy controls that were subjected to OHE under isoflurane anaesthesia and premedicated with acepromazine and methadone. Blood pressure did not differ significantly between the groups’ corresponding phases. Heart rate and end-tidal concentration of isoflurane did not change significantly during the procedure and did not differ significantly between groups.

## References

[1] Egenvall A, Hagman R, Bonnett BN, Hedhammar A, Olson P, Lagerstedt AS (2001). Breed risk of pyometra in insured dogs in Sweden. J Vet Intern Med.

[2] Jitpean S, Hagman R, Ström Holst B, Höglund OV, Pettersson A, Egenvall A (2012). Breed variations in the incidence of pyometra and mammary tumours in Swedish dogs. Reprod Domest Anim.

[3] Gibson A, Dean R, Yates D, Stavisky J (2013). A retrospective study of pyometra at five RSPCA hospitals in the UK: 1728 cases from 2006 to 2011. Vet Rec.

[4] Niskanen M, Thrusfield MV (1998). Associations between age, parity, hormonal therapy and breed, and pyometra in Finnish dogs. Vet Rec.

[5] Hagman R, Lagerstedt AS, Hedhammar A, Egenvall A (2011). A breed-matched case-control study of potential risk-factors for canine pyometra. Theriogenology.

[6] Desborough JP (2000). The stress response to trauma and surgery. Br J Anaesth.

[7] Giannoudis PV, Dinopoulos H, Chalidis B, Hall GM (2006). Surgical stress response. Injury.

[8] Weissman C (1990). The metabolic response to stress: an overview and update. Anesthesiology.

[9] Kataja J, Chrapek W, Kaukinen S, Pimenoff G, Salenius JP (2007). Hormonal stress response and hemodynamic stability in patients undergoing endovascular vs. conventional abdominal aortic aneurysm repair. Scand J Surg.

[10] Yoo KY, Lee MK, Jeong CW, Kim SJ, Jeong ST, Shin MH (2009). Anaesthetic requirement and stress hormone responses in patients undergoing lumbar spine surgery: anterior vs. posterior approach. Acta Anaesthesiol Scand.

[11] Höglund OV, Olsson K, Hagman R, Ohlund M, Olsson U, Lagerstedt AS (2011). Comparison of haemodynamic changes during two surgical methods for neutering female dogs. Res Vet Sci.

[12] Freeman LJ, Rahmani EY, Al-Haddad M, Sherman S, Chiorean MV, Selzer DJ (2010). Comparison of pain and postoperative stress in dogs undergoing natural orifice transluminal endoscopic surgery, laparoscopic, and open oophorectomy. Gastrointest Endosc.

[13] Yoder B, Wolf JS (2005). Canine model of surgical stress response comparing standard laparoscopic, microlaparoscopic, and hand-assisted laparoscopic nephrectomy. Urology.

[14] Ledowski T, Bein B, Hanss R, Paris A, Fudickar W, Scholz J (2005). Neuroendocrine stress response and heart rate variability: a comparison of total intravenous versus balanced anesthesia. Anesth Analg.

[15] Marana E, Scambia G, Maussier ML, Parpaglioni R, Ferrandina G, Meo F (2003). Neuroendocrine stress response in patients undergoing benign ovarian cyst surgery by laparoscopy, minilaparotomy, and laparotomy. J Am Assoc Gynecol Laparosc.

[16] Chernow B, Alexander HR, Smallridge RC, Thompson WR, Cook D, Beardsley D (1987). Hormonal responses to graded surgical stress. Arch Intern Med.

[17] Naitoh T, Garcia-Ruiz A, Vladisavljevic A, Matsuno S, Gagner M (2002). Gastrointestinal transit and stress response after laparoscopic vs conventional distal pancreatectomy in the canine model. Surg Endosc.

[18] Horta RS, Figueiredo MS, Lavalle GE, Costa MP, Cunha RM, Araujo RB (2015). Surgical stress and postoperative complications related to regional and radical mastectomy in dogs. Acta Vet Scand.

[19] Väisänen M, Raekallio M, Kuusela E, Huttunen P, Leppäluoto J, Kirves P (2002). Evaluation of the perioperative stress response in dogs administered medetomidine or acepromazine as part of the preanesthetic medication. Am J Vet Res.

[20] Benson GJ, Grubb TL, Neff-Davis C, Olson WA, Thurmon JC, Lindner DL (2000). Perioperative stress response in the dog: effect of pre-emptive administration of medetomidine. Vet Surg.

[21] Bubalo V, Moens YP, Holzmann A, Coppens P (2008). Anaesthetic sparing effect of local anaesthesia of the ovarian pedicle during ovariohysterectomy in dogs. Vet Anaesth Analg.

[22] Van Goethem B, Van Nimwegen SA, Murell JC, Kirpensteijn J (2012). The effect of neuromuscular blockade on canine laparoscopic ovariectomy: a double-blinded, prospective clinical trial. Vet Surg.

[23] Fox SM, Mellor DJ, Firth EC, Hodge H, Lawoko CR (1994). Changes in plasma cortisol concentrations before, during and after analgesia, anaesthesia and anaesthesia plus ovariohysterectomy in bitches. Res Vet Sci.

[24] Moldal ER, Kristensen AT, Peeters ME, Nodtvedt A, Kirpensteijn J (2012). Hemostatic response to surgical neutering via ovariectomy and ovariohysterectomy in dogs. Am J Vet Res.

[25] Höglund OV, Hagman R, Olsson K, Olsson U, Lagerstedt A-S (2014). Intraoperative changes in blood pressure, heart rate, plasma vasopressin, and urinary noradrenalin during elective ovariohysterectomy in dogs: repeatability at removal of the 1st and 2nd ovary. Vet Surg.

[26] Höglund OV, Hagman R, Stridsberg M (2015). Chromogranin A and cortisol at intraoperative repeated noxious stimuli: surgical stress in a dog model. SAGE Open Med.

[27] Tallant A, Ambros B, Freire C, Sakals S (2016). Comparison of intraoperative and postoperative pain during canine ovariohysterectomy and ovariectomy. Can Vet J.

[28] Hagman R, Reezigt BJ, Bergstrom Ledin H, Karlstam E (2009). Blood lactate levels in 31 female dogs with pyometra. Acta Vet Scand.

[29] Hagman R, Kindahl H, Lagerstedt AS (2006). Pyometra in bitches induces elevated plasma endotoxin and prostaglandin F2α metabolite levels. Acta Vet Scand.

[30] Fransson BA, Lagerstedt AS, Bergstrom A, Hagman R, Park JS, Chew BP (2007). C-reactive protein, tumor necrosis factor alpha, and interleukin-6 in dogs with pyometra and SIRS. J Vet Emerg Crit Care.

[31] Jitpean S, Holst BS, Höglund OV, Pettersson A, Olsson U, Strage E (2014). Serum insulin-like growth factor-I, iron, C-reactive protein, and serum amyloid A for prediction of outcome in dogs with pyometra. Theriogenology.

[32] Jitpean S, Pettersson A, Höglund OV, Holst BS, Olsson U, Hagman R (2014). Increased concentrations of serum amyloid A in dogs with sepsis caused by pyometra. BMC Vet Res.

[33] Jitpean S, Ström-Holst B, Emanuelson U, Höglund OV, Pettersson A, Alneryd-Bull C (2014). Outcome of pyometra in female dogs and predictors of peritonitis and prolonged postoperative hospitalization in surgically treated cases. BMC Vet Res.

[34] Kuida H, Gilbert RP, Hinshaw LB, Brunson JG, Visscher MB (1961). Species differences in effect of gram-negative endotoxin on circulation. Am J Physiol.

[35] Maclean LD, Spink WW, Visscher MB, Weil MH (1956). Studies on the circulatory changes in the dog produced by endotoxin from gram-negative microorganisms. J Clin Invest.

[36] Parratt JR (1973). Myocardial and circulatory effects of *E. coli* endotoxin; modification of responses to catecholamines. Br J Pharmacol.

[37] Fitzmaurice GM, Laird NM, Ware JH (2004). Applied longitudinal analysis.

[38] Littell RC, Milliken GA, Stroup WW, Wolfinger RD, Schabenberger O (2006). SAS for mixed models.

[39] Zilberstein LF, Moens YP, Leterrier E (2008). The effect of local anaesthesia on anaesthetic requirements for feline ovariectomy. Vet J.

[40] Wallace ML, Case JB, Singh A, Ellison GW, Monnet E (2015). Single incision, laparoscopic-assisted ovariohysterectomy for mucometra and pyometra in dogs. Vet Surg.

[41] Adamovich-Rippe KN, Mayhew PD, Runge JJ, Culp WT, Steffey MA, Mayhew KN (2013). Evaluation of laparoscopic-assisted ovariohysterectomy for treatment of canine pyometra. Vet Surg.

[42] Minami S, Okamoto Y, Eguchi H, Kato K (1997). Successful laparoscopy assisted ovariohysterectomy in two dogs with pyometra. J Vet Med Sci.

[43] Aminlashgari N, Höglund OV, Borg N, Hakkarainen M (2013). Degradation profile and preliminary clinical testing of a resorbable device for ligation of blood vessels. Acta Biomater.

[44] Höglund OV, Ingman J, Södersten F, Hansson K, Borg N, Lagerstedt AS (2014). Ligation of the spermatic cord in dogs with a self-locking device of a resorbable polyglycolic based co-polymer—feasibility and long-term follow-up study. BMC Res Notes.

[45] da Mota Costa MR, de Abreu Oliveira AL, Ramos RM, de Moura Vidal LW, Borg N, Höglund OV (2016). Ligation of the mesovarium in dogs with a self-locking implant of a resorbable polyglycolic based co-polymer—a study of feasibility and comparison to suture ligation. BMC Res Notes.

[46] Merin RG, Bernard JM, Doursout MF, Cohen M, Chelly JE (1991). Comparison of the effects of isoflurane and desflurane on cardiovascular dynamics and regional blood flow in the chronically instrumented dog. Anesthesiology.

[47] Zbinden AM, Petersen-Felix S, Thomson DA (1994). Anesthetic depth defined using multiple noxious stimuli during isoflurane/oxygen anesthesia. II. Hemodynamic responses. Anesthesiology.

[48] Brearley JC, Heath SE, Jones RS, Skerritt GC, Bishop Y (2001). Drugs acting on the nervous system. The veterinary formulary.

[49] Ruffato M, Novello L, Clark L (2015). What is the definition of intraoperative hypotension in dogs? Results from a survey of diplomates of the ACVAA and ECVAA. Vet Anaesth Analg.

[50] Ingvast-Larsson C, Holgersson A, Bondesson U, Lagerstedt AS, Olsson K (2010). Clinical pharmacology of methadone in dogs. Vet Anaesth Analg.

[51] Hashem A, Kietzmann M, Scherkl R (1992). The pharmacokinetics and bioavailability of acepromazine in the plasma of dogs. Deutsch Tierarztl Wochenschr.

[52] Baldini G, Bagry H, Carli F (2008). Depth of anesthesia with desflurane does not influence the endocrine-metabolic response to pelvic surgery. Acta Anaesthesiol Scand.

[53] Boström IM, Nyman GC, Lord PE, Häggström J, Jones BE, Bohlin HP (2002). Effects of carprofen on renal function and results of serum biochemical and hematologic analyses in anesthetized dogs that had low blood pressure during anesthesia. Am J Vet Res.

[54] Frendin JH, Boström IM, Kampa N, Eksell P, Häggström JU, Nyman GC (2006). Effects of carprofen on renal function during medetomidine-propofol-isoflurane anesthesia in dogs. Am J Vet Res.

[55] Howe LM (2006). Surgical methods of contraception and sterilization. Theriogenology.

[56] Meurs KM, Miller MW, Slater MR (1996). Comparison of the indirect oscillometric and direct arterial methods for blood pressure measurements in anesthetized dogs. J Am Anim Hosp Assoc.

[57] Sawyer DC, Brown M, Striler EL, Durham RA, Langham MA, Rech RH (1991). Comparison of direct and indirect blood pressure measurement in anesthetized dogs. Lab Anim Sci.

[58] Bodey AR, Michell AR, Bovee KC, Buranakurl C, Garg T (1996). Comparison of direct and indirect (oscillometric) measurements of arterial blood pressure in conscious dogs. Res Vet Sci.

[59] Valverde A, Gianotti G, Rioja-Garcia E, Hathway A (2012). Effects of high-volume, rapid-fluid therapy on cardiovascular function and hematological values during isoflurane-induced hypotension in healthy dogs. Can J Vet Res.

[60] Wagner AE, Worland GA, Glawe JC, Hellyer PW (2008). Multicenter, randomized controlled trial of pain-related behaviors following routine neutering in dogs. J Am Vet Med Assoc.

[61] Höglund OV. Inventor Tissue ligation device. US patent 8696692; 2014.

[62] Höglund OV. Inventor device and method for tissue ligation—patent application. US patent 20150157327; 2015.

